# Patient-Reported Influence of Sociopolitical Issues on Post-*Dobbs* Vasectomy Decisions

**DOI:** 10.1001/jamanetworkopen.2024.54430

**Published:** 2025-01-10

**Authors:** Lucille G. Cheng, Kari White, Marian Jarlenski, Kathleen Hwang

**Affiliations:** 1 University of Pittsburgh School of Medicine, Pittsburgh, Pennsylvania; 2 Resound Research for Reproductive Health, Austin, Texas; 3 Department of Health Policy and Management and Center for Innovative Research on Gender Health Equity, University of Pittsburgh, Pittsburgh, Pennsylvania; 4 Department of Surgery, Division of Urology, Perelman School of Medicine at the University of Pennsylvania, Philadelphia

## Abstract

This cross-sectional study of US patients initiating a vasectomy procedure examines the association of vasectomy decision-making with sociopolitical issues following the US Supreme Court’s repeal of the right to abortion.

## Introduction

Recent reproductive health care policy discourse has predominantly centered female contraception.^1^ However, the potential spillover effects of female-targeted policy changes on male contraception motivations are understudied. Following the June 2022 *Dobbs v Jackson* decision overturning the constitutional right to abortion recognized since *Roe v Wade*, vasectomy interest and total procedures performed increased.^2,3^ In this study of patients, we explore the association of sociopolitical issues with patients’ vasectomy timing and decision-making following *Dobbs*.

## Methods

In this cross-sectional study, we surveyed patients in Pittsburgh, Pennsylvania, who had initiated a vasectomy after June 2022. Surveys were administered between June 2023 and May 2024 (eMethods in Supplement 1). Patients were approached either prospectively and in-person at appointment time or retrospectively over the phone. The University of Pittsburgh institutional review board approved the study protocol. Informed consent was acquired both verbally and in writing for in-person and verbally for over the phone. We followed both the Consensus-Based Checklist for Reporting of Survey Studies (CROSS) and American Association for Public Opinion Research (AAPOR) reporting guidelines.

Patient demographic characteristics, factors influencing vasectomy, and general economic and political interest data were collected via modified Likert and free-text questions (eAppendix in Supplement 1). Race was self-reported via patients’ electronic health records and included Asian, Black, Hispanic or Latino, White, and other. Multivariable logistic regression and nonparametric testing (ie, Mann-Whitney *U*, Kruskal-Wallis) was applied, as appropriate, using Stata/SE version 18 (StataCorp). Two-sided *P* < .05 was considered statistically significant.

## Results

Of 469 patients approached, we received 315 surveys and included 311 responses (251 married [81.0%]; 287 privately insured [92.3%]), yielding a response rate of 67%. Participants had a median (IQR) age of 37.7 (33.8-41.8) years and had a median (IQR) of 2 (1-2) biological children (Table 1). Overall, 59 respondents (19.0%) did not have children (ie, child-free).

**Table 1.** Survey Respondent Demographics and Analyses of Sociopolitical Influence

Characteristics	Patients, No. (%)		*P* value
	Total (N = 311)	Influenced by sociopolitical factors (n = 95)	
Age, y			
≤30	43 (13.8)	16 (16.8)	.01
31-35	67 (21.5)	28 (29.5)	
36-40	108 (34.7)	27 (28.4)	
41-45	67 (21.5)	22 (23.2)	
≥46	26 (8.4)	2 (2.1)	
Marital status			
Married, not separated	251 (81.0)	63 (66.3)	<.001^a^
Married, separated	3 (1.0)	1 (1.1)	
Widowed	3 (1.0)	1 (1.1)	
Divorced	12 (3.8)	4 (4.2)	
Single	30 (9.6)	21 (22.1)	
Other	11 (3.6)	5 (5.3)	
Biological children, total			
0	58 (18.7)	39 (41.5)	<.001^b^
1	39 (12.6)	17 (18.1)	
2	141 (45.5)	26 (27.7)	
3	60 (19.4)	10 (10.6)	
≥4	12 (3.8)	2 (2.1)	
Residence			
In Allegheny County	221 (71.1)	74 (77.9)	.35
In Pennsylvania, outside Allegheny County	80 (25.7)	18 (5.8)	
Outside Pennsylvania	10 (3.2)	3 (3.2)	
Race and ethnicity			
Asian	7 (2.3)	2 (2.1)	.43
Black	18 (5.8)	9 (9.5)	
Hispanic or Latino	13 (4.2)	7 (7.4)	
White (non-Hispanic)	272 (90.6)	77 (81.1)	
Other	3 (1.0)	0	
Insurance status			
Private insurance	287 (92.3)	87 (91.6)	.44
Public insurance (ie, Medicaid)	22 (7.1)	7 (7.4)	
Uninsured	1 (0.3)	1 (1.1)	
Other	1 (0.3)	0	
Highest level of education completed			
High school, equivalent (ie, GED), or less	24 (7.7)	9 (9.5)	.45
Some college but no degree	43 (13.8)	16 (16.8)	
Associate’s degree	37 (11.9)	15 (15.8)	
Bachelor’s degree	126 (40.5)	37 (39.0)	
Master’s degree	45 (14.5)	10 (10.5)	
Doctorate	35 (11.3)	8 (8.4)	
Other	1 (0.3)	0	
Voter registration status			
Registered	278 (90.6)	89 (94.7)	.06
Not registered	18 (5.9)	5 (5.3)	
Not sure	2 (0.6)	0	
Not applicable	9 (2.9)	0	
Voted in 2020 presidential election			
Yes	266 (86.6)	84 (89.4)	.30
No	30 (9.8)	9 (9.6)	
Not sure	1 (0.3)	0	
Not applicable	10 (3.3)	1 (1.1)	
Voted in 2022 local election			
Yes	207 (68.3)	67 (73.6)	.19
No	83 (27.4)	21 (23.1)	
Not sure	2 (0.7)	1 (1.1)	
Not applicable	11 (3.6)	2 (2.2)	

Abbreviation: GED, General Education Development test.

^a^ Ad hoc analysis on a sublevel pairwise basis was performed using Dunn test with Bonferroni adjustment. The analysis reported is a comparison between single marital status and married, not separated.

^b^ Ad hoc analysis on a sublevel pairwise basis was performed using Dunn test with Bonferroni adjustment. The analysis reported is a comparison between 2 or 3 children and zero children, respectively (both *P* < .001).

When asked to rank the importance of factors influencing vasectomy, 95 respondents (30.5%) answered “sociopolitical issues (ie, reversal of *Roe v Wade*)” were an “important” or “the most important” factor in their vasectomy decision (Table 2). Among the entire cohort, nonparametric analyses demonstrated that respondents who reported that sociopolitical factors influenced their decision were more often younger (eg, ages 31 to 35 years, 28 of 95 [29.5%] vs 39 of 216 [18.1%]), child-free (39 of 95 [41.5%] vs 19 of 216 [8.8%]), or unmarried (single status, 21 of 95 [22.1%] vs 9 of 216 [4.2%]) (Table 1). Multivariable-adjusted logistic regression demonstrated child-free men had 5 times greater odds to indicate sociopolitical issues influenced their vasectomy decision compared with those with children (OR, 5.5; 95% CI, 2.7-10.9).

**Table 2.** Participants’ Ranking of Factors Influencing Vasectomy Decision

Factor^a^	No. of respondents^b^	Patients, No. (%)	
		Important or one of the most important factors	Minor factor or did not factor
Close friend/family member received a vasectomy and spoke positively about the experience	310	132 (43)	178 (57)
Positive support from spouse/partner	310	280 (90)	30 (10)
Quick recovery time	309	205 (66)	104 (34)
High contraceptive effectiveness	310	303 (98)	7 (2)
Comparative safety and lower costs associated with vasectomy compared with female sterilization	310	263 (85)	47 (15)
Sociopolitical issues (ie, reversal of *Roe v Wade*)	310	95 (31)	215 (69)
Economic issues (ie, inflation, rising costs)	310	89 (29)	221 (71)
Climate change/increasing population burden (ie, “enough people on the planet”)	310	63 (20)	247 (80)
Other^c^	49	46 (94)	3 (6)

^a^ Factors reported verbatim from the survey to represent what participants saw when they were ranking.

^b^ One participant did not answer any of these questions. Another participant did not rank “quick recovery time” in their response.

^c^ Other included space for a free-text response. Factors listed by respondents included: wife’s health, ie, difficult past pregnancies or birth control adverse effects; patient self-perceived old age; quality of life for future offspring; family completeness; specific personal experiences, ie, divorce, close friends having unplanned pregnancies; knowledge that vasectomy would not affect hormone levels or semen.

Men spent a mean (SD) 2.8 (3.7) years considering vasectomy before deciding, with those indicating sociopolitical factors as an important factor spending 2.5 years longer on average considering before deciding compared with those who indicated sociopolitical factors were not a factor (mean [SD] time considering vasectomy: 55.8 [77.4] months vs 25.4 [24.2] months; *P* = .005). Subgroup analysis demonstrated child-free men spent longer considering vasectomy than those with children (54.5 months [95% CI, 38.0-71.0 months] vs 28.0 months [95% CI, 23.4-32.7 months], respectively; *P* < .001).

## Discussion

Nearly one-third of participants indicated sociopolitical issues influenced their vasectomy decision, despite the fact these policies have targeted female reproductive policy. These patient-reported motivations are consistent with recent research using administrative data that found a rise in vasectomy procedure volume after the *Dobbs* decision.^2,3,4,5^

While increases in child-free men or younger men pursuing vasectomy following *Dobbs* has led to some concern around sterilization regret,^6^ our data demonstrate patients spent a significant time considering vasectomy and are unlikely to be making hasty decisions solely motivated by sociopolitical trends. These findings further support the notion that the *Dobbs* decision may have instilled a newfound sense of urgency for vasectomy follow-through for men who had already been considering the procedure, as has also been hypothesized in a 2023 study.^4^

Study limitations include cross-sectional data and single-site recruitment from southwestern Pennsylvania, which may limit generalizability. Additionally, our sample is largely homogenous in racial characteristics and high levels of political engagement per voting behavior, which may skew findings; more information about motivations in other populations is warranted.

Despite having no direct impact to male contraception, social policy factors, including abortion bans, may play a role in men’s reproductive decision-making. Our study found that these policies are associated with changes in contraceptive decision-making that are not isolated to people who can become pregnant.

## Notes

Supplement 1.
eMethods.
**eAppendix.** Patient Survey

Supplement 2.
Data Sharing Statement
