# Error in Results

**DOI:** 10.1001/jamanetworkopen.2025.0785

**Published:** 2025-02-24

In the Research Letter “Patient-Reported Influence of Sociopolitical Issues on Post-*Dobbs* Vasectomy Decisions,”^1^ published January 10, 2025, there were errors in demographic data compared in the Results. The title of Table 1 has also been changed to clarify the comparison. The article has been corrected.^1^
