# Supplementary material for: Patient-Reported Influence of Sociopolitical Issues on Post-Dobbs Vasectomy Decisions
Source: JAMA Netw Open. 2025 Jan 10;8(1):e2454430. doi: 10.1001/jamanetworkopen.2024.54430 (PMC11724337; doi:10.1001/jamanetworkopen.2024.54430)
Supplement: Supplement 1. — eMethods. eAppendix. Patient Survey [file jamanetwopen-e2454430-s001.pdf]

Supplemental Online Content

Cheng LG, White K, Jarlenski M, Hwang K. Patient-reported influence of sociopolitical issues on post-*Dobbs* vasectomy decisions. *JAMA Netw Open.* 2025;8(1):e2454430. doi:10.1001/jamanetworkopen.2024.54430

eMethods.

eAppendix. Patient Survey

This supplemental material has been provided by the authors to give readers additional information about their work.

© 2025 Cheng LG et al. *JAMA Network Open*.

eMethods

Participants and Procedures

This cross-sectional survey study was conducted at a male reproductive health outpatient clinic within a large academic healthcare system in Southwestern Pennsylvania. Participants were eligible for the study if they were 18 years or older and had initiated either a vasectomy consultation or appointment after June 2022. The survey was administered between June 2023 and May 2024. Participants were either approached prospectively and in-person at the time of their appointment or retrospectively over the phone. Retrospective participants were chosen based on recency of their procedure to reduce recall bias and minimize the time passed between date of the patient's appointment and date of outreach, with a maximum of six months. Given current vasectomy prevalence data of 5% and the adult male population of Southwestern Pennsylvania, our target sample size was 298 patients with a 95% level of confidence based on margins of errors around prevalence estimates.[15,16] The institutional review board of the University of Pittsburgh reviewed and approved the study protocol. No incentive was provided to study participants. This study followed the Consensus-Based Checklist for Reporting of Survey Studies (CROSS) guidelines.[17] Instrument pilot testing was conducted by administering the survey in-person or over the phone to four men who had recently begun the process for or received a vasectomy.

Measures

Demographic data, obtained through self-report or through patients' electronic medical record, included race, type of insurance coverage, date of birth, zip code of home residence, marital/partner status and length of relationship (if applicable), highest level of education attained, number of biological children, and their age and sex (if applicable).

Three open-ended questions gauged vasectomy knowledge and general interest. Vasectomy knowledge was assessed by a question that asked, "To the best of your knowledge, when did you first learn about vasectomies (approximate date or year)?" Time spent considering a vasectomy was assessed by asking, "Approximately how long had you been thinking about getting a vasectomy before you decided to receive one?" Inciting factors influencing vasectomy interest was gauged by asking, "What prompted your interest in receiving a vasectomy?"

The influence of eight personal, procedural, and societal factors influencing vasectomy were assessed with the following question, "Thinking about your decision to pursue a vasectomy, how important to you was each of the following?" Factors were selected based on their usage in existing vasectomy literature and clinical expertise.[18-21] They were as follows: (1) "close friend or

© 2025 Cheng LG et al. *JAMA Network Open*.

family member received a vasectomy and spoke positively about the experience"; (2) "positive support from spouse or partner"; (3) "quick recovery time"; (4) "high contraceptive effectiveness"; (5) "comparative safety and lower costs associated with vasectomy compared to female sterilization"; (6) "sociopolitical issues (i.e. reversal of Roe v. Wade)"; (7) "economic issues (i.e. inflation and rising costs)"; and (8) "climate change or increasing population burden (i.e. "enough people on the planet")". Options included "Most important factor," "Important factor, but not the most important," "A minor factor," or "Did not factor into decision." A factor which received an answer of "most important factor" or "important factor, but not the most important" was deemed to be influential in the patient's vasectomy decision, whereas "a minor factor" or "did not factor into decision" was deemed as not influential in the patient's decision.

General interest in sociopolitical and economic issues was gauged through voter registration status and voting in the most recent elections. This was ascertained by asking participants: "Are you registered to vote?", "Did you vote in the 2020 presidential election?", and "Did you vote in your local 2022 election?". Degree of importance of various socioeconomic and political issues was assessed with the following question: "Thinking about the country as a whole, how would you classify the following problems?" Participants who responded "A very big problem" or "A moderately big problem" were classified as having found that issue large, and those who responded "A small problem" or "Not a problem at all" were classified as not having found that issue large. The same question was also asked about their home residence state.

Patient knowledge and beliefs about the impact that reproductive healthcare legislation had on male reproductive healthcare options was assessed by asking, "To the best of your knowledge, how does the overturning of Roe v. Wade impact your access to reproductive healthcare across the state of Pennsylvania?" Reproductive healthcare options assessed included in vitro fertilization (IVF), sperm banking or cryopreservation, hormone therapy (i.e. testosterone replacement), and vasectomy. Possible responses included "Prevent access," "Limit access", or "No change in access."

Data Analysis

Statistical analysis was performed from March to May 2024 using STATA SE, version 18.0 (StataCorp LLC, College Station, TX). Data cleaning yielded four responses where 50% or less of the survey had been completed; these responses were removed from analysis. Significance level was set at a two-sided P<.05. Descriptive statistics were reported with means (SD) or frequencies (percentages) as relevant. Given the non-normal variable distributions of our data, nonparametric testing was applied as appropriate (i.e. Mann-Whitney *U* or Kruskal-Wallis tests

© 2025 Cheng LG et al. *JAMA Network Open*.

for cohort and demographic comparison, multivariable logistic regression). We further compared two cohorts of clinical interest, men without biological children and men 30 years of age and younger, to their counterparts (men with biological children and men older than 30, respectively), due to historical concerns about vasectomy regret in these populations. We subsequently created a multivariable logistic regression model evaluating the impact of having no biological children had on the odds of sociopolitical issues being influential, controlling for decision length, age, and marital status.

© 2025 Cheng LG et al. *JAMA Network Open*.

eAppendix. Patient survey

Magee-Womens Hospital

Of UPMC Health System

300 Halket Street
Pittsburgh, PA 15213-3180

Participant Survey

PLEASE REVIEW AND COMPLETE THE INFORMATION BELOW:

Participant ID: __________________________ Date of birth: _________________________________
Home zip code: ________________________

Marital status (choose one):

- ❑ Married (not separated)
- ❑ Married (separated)
- ❑ Widowed
- ❑ Divorced
- ❑ Single
- ❑ Other

How long have you been with your current partner (i.e. 6 months, 2 years)? __________________

Highest level of schooling completed:

- ❑ Middle school
- ❑ High school diploma or equivalency (GED)
- ❑ Some college but no degree
- ❑ Associate's degree
- ❑ Bachelor's degree
- ❑ Master's degree
- ❑ Doctorate
- ❑ Other
- ❑

Do you have any biological children? (please select one): Y / N
If yes, how many? ___________
If yes, what are their current ages and sex?

Child #	Sex	Months	Years
1			
2			
3			
4			

© 2025 Cheng LG et al. *JAMA Network Open*.

FOR EACH OF THE FOLLOWING STATEMENTS, PLEASE COMPLETE TO THE BEST OF YOUR ABILITY.

Date of vasectomy consultation: ____

Date of vasectomy appointment: ____

To the best of your knowledge, when did you first learn about vasectomies (approximate date or year)?
____

____

Approximately how long had you been thinking about getting a vasectomy before you decided to receive one? ____

____

What prompted your interest in receiving a vasectomy? ___________________________________

____

____

____

____

____

© 2025 Cheng LG et al. *JAMA Network Open*.

FOR EACH OF THE FOLLOWING, PLEASE SELECT YES IF THE STATED FACTOR INFLUENCED YOUR DECISION TO RECEIVE A VASECTOMY.

Thinking about **your decisions to pursue a vasectomy**, how important to you was each of the following?

	Most important factor	Important factor, but not the most important	A minor factor	Did not factor into decision
Close friend / family member received a vasectomy and spoke positively about the experience	☐	☐	☐	☐
Positive support from spouse / partner	☐	☐	☐	☐
Quick recovery time	☐	☐	☐	☐
High contraceptive effectiveness	☐	☐	☐	☐
Comparative safety and lower costs associated with the vasectomy compared to female sterilization	☐	☐	☐	☐
Sociopolitical issues (i.e. reversal of Roe v. Wade)	☐	☐	☐	☐
Economic issues (i.e. inflation and rising costs)	☐	☐	☐	☐
Climate change / increasing population burden (i.e. "enough people on the planet")	☐	☐	☐	☐
Other (Please elaborate: ________________________ ________________________ ________________________)	☐	☐	☐	☐

© 2025 Cheng LG et al. *JAMA Network Open*.

THE FOLLOWING QUESTIONS WILL ASK ABOUT YOUR SOCIOECONOMIC AND POLITICAL INTEREST AND INVOLVEMENT.

1. Are you a registered voter?

Yes	No	Not sure	Not applicable

2. Did you vote in the 2020 presidential election?

Yes	No	Not sure	Not applicable

Thinking about **the country as a whole**, how would you classify the following problems?

	A very big problem	A moderately big problem	A small problem	Not a problem at all
Inflation	☐	☐	☐	☐
Unemployment	☐	☐	☐	☐
Affordability of health care	☐	☐	☐	☐
Reproductive rights	☐	☐	☐	☐
Other (Describe: ___)	☐	☐	☐	☐

3. Did you vote in the 2022 local election?

Yes	No	Not sure	Not applicable

Thinking about **your home residence state,** how would you classify the following problems?

	A very big problem	A moderately big problem	A small problem	Not a problem at all
Inflation	☐	☐	☐	☐
Unemployment	☐	☐	☐	☐
Affordability of health care	☐	☐	☐	☐
Reproductive rights	☐	☐	☐	☐
Other (Describe: ___)	☐	☐	☐	☐

© 2025 Cheng LG et al. *JAMA Network Open*.

4. To the best of your knowledge, how does the overturning of Roe v. Wade impact **your access to reproductive healthcare** across the state of Pennsylvania?

	Prevent access	Limit access	No change in access
In vitro fertilization	☐	☐	☐
Sperm banking / cryopreservation	☐	☐	☐
Hormone therapy (i.e. testosterone replacement)	☐	☐	☐
Vasectomy	☐	☐	☐
Other (Please elaborate: ______________________)	☐	☐	☐

END SURVEY

Thank you for completing this survey.

We may conduct follow up interviews with participants to discuss survey responses. If you are open to being contacted to participate in an interview, please check the following box. Interview participants will be compensated for their time.

☐ I consent to being contacted for a follow-up interview.

Name:________________________ **Date: __________________________**

Signature: ____

© 2025 Cheng LG et al. *JAMA Network Open*.
