# Supplementary material for: Patient-Reported Influence of Sociopolitical Issues on Post-Dobbs Vasectomy Decisions
Source: JAMA Netw Open. 2025 Jan 10;8(1):e2454430. doi: 10.1001/jamanetworkopen.2024.54430 (PMC11724337; doi:10.1001/jamanetworkopen.2024.54430)
Supplement: Supplement 2. — Data Sharing Statement [file jamanetwopen-e2454430-s002.pdf]

Data Sharing Statement

Cheng. Patient-Reported Influence of Sociopolitical Issues on Post-Dobbs Vasectomy Decisions. *JAMA Netw Open*. Published January 10, 2025.
doi:10.1001/jamanetworkopen.2024.54430

Data
Data available: No
